# Oral health-related quality of life in cardiovascular patients- a systematic review

**DOI:** 10.1186/s12872-026-05564-8

**Published:** 2026-02-05

**Authors:** Kamyar Nasiri, Farzaneh Nikfarjam, Ali keshavarzian, Naghmeh shenasa, Wissam Hamid Al-janabi, Radhwan Abdul Kareem, Hayder Naji Sameer, Fateme Moradi

**Affiliations:** 1 Department of dentistry, Islamic Azad University of Medical Sciences, Tehran, Iran; 2 Shiraz University of Medical Sciences, Shiraz, Iran; 3 Golestan University of Medical Sciences, Gorgan, Iran; 4 Department of Endodontics, Shahrekord University of Medical Sciences, Shahrekord, Iran; 5 Department of Dentistry, Al-Zahrawi University College, Karbala, Iraq; 6 Ahl Al Bayt University, Kerbala, Iraq; 7 Collage of Pharmacy, National University of Science and Technology, Dhi Qar, 64001 Iraq

**Keywords:** Oral health, Cardiovascular diseases, Oral health-related quality of life, Systematic review

## Abstract

**Objectives:**

This systematic review aimed to synthesize evidence from observational studies on oral health-related quality of life (OHRQoL) among patients with cardiovascular diseases (CVDs), addressing the lack of comprehensive data on the determinants of impaired OHRQoL in this population.

**Methods:**

A comprehensive and unrestricted search was conducted in PubMed, Scopus, and Web of Science from inception to July 2025 to identify cross-sectional, case-control, and cohort studies reporting on OHRQoL in cardiovascular patients. Two independent reviewers performed study screening and data extraction. English-language full-text articles were included. The review protocol was registered in PROSPERO (CRD42024609168). The methodological quality of included studies was assessed using the Newcastle–Ottawa Scale (NOS). The search strategy included the keywords: “cardiovascular disease” AND “oral-health-related quality of life. “Eligible studies involved adult patients (aged 18 years or older) and reported outcomes using validated OHRQoL instruments.

**Results:**

Out of 327 records initially identified, 23 observational studies met the inclusion criteria. Overall, most studies were of moderate to high methodological quality. These studies involved diverse cardiovascular patient populations—including those with stroke, heart failure, and general CVD—with sample sizes ranging from 27 to 7,702. OHRQoL was primarily assessed using OHIP-14 and GOHAI. Mean OHRQoL scores showed substantial variability across studies (OHIP-14 range: 2.9–33.0; GOHAI range: 15.2–51.9). Across the majority of studies, patients with cardiovascular diseases—particularly those with stroke and heart failure—demonstrated poorer OHRQoL compared with reference or less severe groups.

**Conclusion:**

Oral health significantly influences quality of life among individuals with cardiovascular conditions. Clinicians should prioritize oral health assessment and preventive care, particularly for vulnerable groups such as older stroke survivors. Future research should explore targeted interventions to improve OHRQoL in these populations.

**Supplementary Information:**

The online version contains supplementary material available at 10.1186/s12872-026-05564-8.

## Introduction

Cardiovascular diseases (CVDs) remain among the leading causes of mortality, disability, and healthcare burden worldwide, accounting for nearly one-third of all global deaths annually [1, 2]. The direct healthcare costs attributable to CVDs exceed those of many other disease groups, imposing a substantial economic burden on health systems globally [3]. Beyond their clinical outcomes, CVDs are associated with significant physical, psychological, and socioeconomic consequences that adversely affect patients’ health-related quality of life compared with healthy populations [4]. Although advances in medical care have substantially improved survival and life expectancy, they have also increased the prevalence of chronic cardiovascular conditions, thereby shifting clinical priorities toward outcomes beyond survival, particularly quality of life, as a central component of chronic disease management [1, 2].

Quality of life is a multidimensional construct influenced by various health-related factors, among which oral health plays a critical role. Oral health affects well-being, life satisfaction, and activities of daily living (ADL), and its impact becomes more pronounced in individuals with chronic diseases [5, 6]. The World Health Organization has emphasized that oral health will become an even more urgent concern as the global population ages [7]. Importantly, even among patients with potentially life-threatening conditions, oral health remains an essential component of comprehensive patient care and overall quality of life [8, 9].

Patients with cardiovascular diseases may be particularly vulnerable to oral health problems. Long-term use of cardiovascular medications including calcium channel blockers, diuretics, beta-blockers, and angiotensin-converting enzyme inhibitors has been associated with oral complications such as lichenoid reactions, angioedema, and reduced salivary flow [10, 11]. In addition, epidemiological studies have demonstrated strong associations between poor oral health and both cerebrovascular events and cardiovascular disease [12, 13]. Tooth loss, in particular, has emerged as a prominent oral health indicator related to heart failure; a large prospective cohort study in Australia reported that individuals with complete tooth loss had approximately twice the risk of developing heart failure compared with those retaining 20 or more natural teeth [14]. Other oral health conditions including edentulousness, soft tissue lesions, xerostomia-related pain, ill-fitting prostheses, eating and drinking restrictions, and oral infections may further compromise overall health and quality of life in this population [15].

To capture the subjective and functional impact of oral conditions on daily life, the concept of oral health-related quality of life (OHRQoL) has been increasingly adopted over the past two decades [16]. OHRQoL reflects the interrelationship between oral health, general health status, and overall quality of life, emphasizing patients’ perceptions of how oral conditions affect their physical, psychological, and social functioning [17–20]. Historically, the psychosocial consequences of oral diseases received limited attention because such conditions are rarely life-threatening; however, accumulating evidence since the late 1970 s has highlighted their substantial impact on social roles and daily activities, leading to the development of the OHRQoL construct [21].

Assessing OHRQoL in patients with cardiovascular diseases is therefore particularly important, as these individuals often experience chronic, complex health conditions that may uniquely influence their oral health and its consequences [22].

Although several studies have reported impaired OHRQoL among patients with CVD, findings remain heterogeneous due to differences in study populations, inclusion criteria, clinical characteristics, and OHRQoL assessment instruments [23–26]. Moreover, disease-specific factors such as long-term medication use, physical limitations, and dietary restrictions may further distinguish OHRQoL in cardiovascular patients from that of other populations. Despite the importance of understanding this relationship, there is a lack of comprehensive systematic reviews that explore the full extent of OHRQoL impairments in this population. This gap in the literature highlights the need for a more thorough synthesis of existing studies, as current research has yet to fully examine the complex interplay between oral health and cardiovascular conditions. Given the biological and psychosocial factors at play, this systematic review aims to synthesize existing evidence on oral health-related quality of life (OHRQoL) in patients with cardiovascular diseases, identify the key oral health conditions associated with impaired OHRQoL, and examine patterns across different cardiovascular populations and assessment instruments. Addressing this gap will better inform clinical practices and interventions aimed at improving the overall health and well-being of these patients.

## Method

This systematic review was conducted and reported according to the PRISMA 2020 guidelines. The protocol was registered on the International Prospective Register of Systematic Reviews (PROSPERO) on November 14, 2024 (registration number: CRD42024609168) [27].

### Search strategy

As this systematic review aimed to describe and assess oral health–related quality of life (OHRQoL) in patients with cardiovascular diseases, the review question was descriptive rather than interventional. Therefore, the search strategy was developed using the Population (patients with cardiovascular diseases) and Outcome (OHRQoL) components of the PICO framework, while Intervention and Comparator were not applicable.

In accordance with the registered protocol, the search strategy was predefined, and a comprehensive search of electronic databases, including PubMed, Scopus, and Web of Science, was conducted from database inception and updated on 15 July 2025 to include studies published up to that date. No language or publication-type restrictions were applied during the database search.

No language or publication-type restrictions were applied during the database search. However, during the study selection stage, eligibility was limited to English-language publications to ensure accuracy and consistency in data extraction and quality assessment, given resource limitations for translation.

Eligibility criteria.

#### Inclusion criteria

- Observational studies with cross-sectional, case-control, or cohort designs.
- Studies involving adults (≥ 18 years) with a confirmed diagnosis of cardiovascular diseases (e.g., coronary artery disease, heart failure, stroke, etc.).
- Studies that report quantitative assessment of oral health-related quality of life (OHRQoL) using validated instruments, such as OHIP, GOHAI, or other standardized tools.
- Studies published in peer-reviewed journals, written in English, and published up to July 2025.

#### Exclusion criteria

- Intervention studies, such as randomized controlled trials, quasi-experimental studies, and studies assessing treatment effects (focus is on observational assessment of OHRQoL).
- Reviews (systematic, narrative), conference abstracts, editorials, letters to the editor, and gray literature (e.g., theses, dissertations, preprints).
- Non-English publications or studies for which the full text was not accessible.
- Randomized controlled trials and other interventional studies, including those reporting baseline (pre-intervention) OHRQoL data, were excluded to ensure methodological homogeneity.

### Study selection process

All retrieved records were imported into EndNote 21 for reference management, and duplicates were removed. Formal inter-rater reliability statistics (e.g., Cohen’s kappa) were not calculated. Given the relatively limited number of retrieved records and the use of full independent dual screening for all stages, discrepancies were resolved through discussion and consensus, consistent with PRISMA 2020 recommendations emphasizing transparency over mandatory agreement statistics. Two reviewers independently screened the titles and abstracts for potential relevance. Subsequently, the full texts of potentially eligible studies were retrieved and assessed independently against the predefined inclusion and exclusion criteria.

**Fig. 1:**
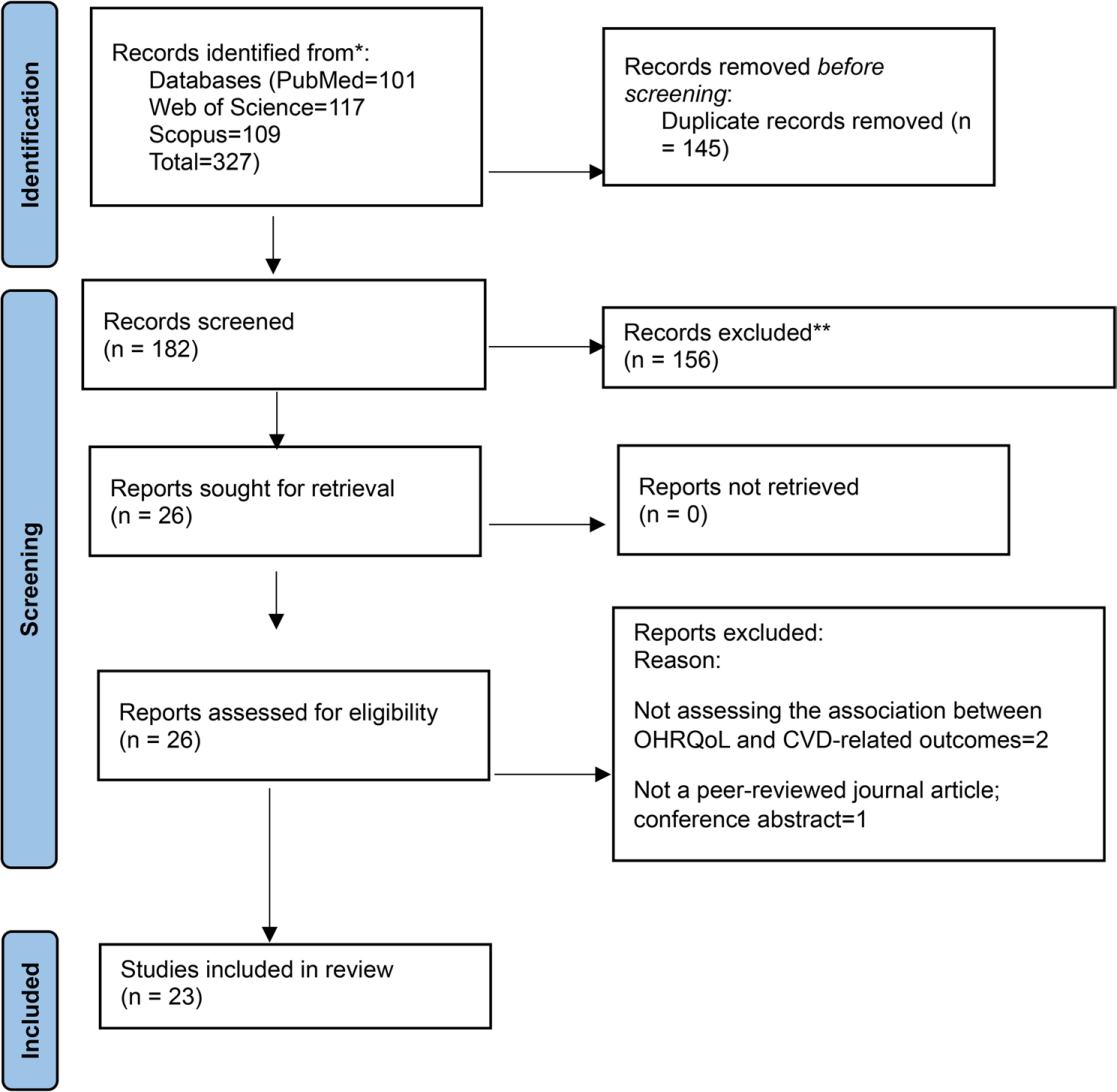
Flow diagram of records identified through database searching

Any disagreements at any stage of the screening process were resolved through discussion, and when necessary, consultation with a third reviewer. The study selection process is presented using a PRISMA flow diagram (Fig. 1).

### Data extraction

Two reviewers independently screened the titles and abstracts for relevance. The following data were collected:

- Study characteristics: authors, publication year, country, study design, sample size, participants’ mean age.
- Clinical details: type of cardiovascular disease, assessment tools used for OHRQoL.
- Key findings and OHRQoL outcomes.
- Methodological quality indicators and reliability of tools used.

OHRQoL in patients with cardiovascular diseases was assessed using standardized questionnaires, such as the Geriatric Oral Health Assessment Index (GOHAI) and the Oral Health Impact Profile (OHIP), along with factors related to oral health conditions that may influence OHRQoL. For OHRQoL assessment tools, data extraction also included the specific instrument used, the language and culturally validated version applied (e.g., Chinese, Persian, Romanian), and any reported psychometric properties, such as internal consistency (Cronbach’s α). When validation details were not explicitly reported by the original studies, this was documented accordingly.

### Quality assessment

The methodological quality of the included cohort and case-control studies was assessed using the Newcastle–Ottawa Scale (NOS) [28], which evaluates studies across three domains: selection of participants, comparability of study groups, and ascertainment of exposure or outcome.

For cross-sectional studies, an adapted version of the Newcastle–Ottawa Scale proposed by Carra et al. was used [29]. This adapted tool assesses study quality across comparable domains and yields a total score ranging from 0 to 9 points. Each study was independently assessed by two reviewers, and discrepancies were resolved through discussion to reach consensus. Based on the total score, studies were categorized as high quality (7–9 points), moderate/fair quality (4–6 points), or low quality (0–3 points).

## Results

### Study selection

A total of 327 records were identified through electronic database searching. After removal of duplicates, 182 unique articles remained. Screening of titles and abstracts led to the exclusion of 156 records. Of the 26 full-text articles assessed for eligibility, three were excluded, two did not evaluate the association between oral health-related quality of life (OHRQoL) and cardiovascular disease (CVD)-related outcomes, and one was a conference abstract rather than a peer-reviewed article. Consequently, 23 studies were included in the final synthesis. The study selection process is presented in the PRISMA flow diagram (Fig. 1).

### Methodological quality of included studies

Different versions of the NOS were applied depending on study design, including the adapted NOS for cross-sectional studies. Quality assessment using the Newcastle–Ottawa Scale (NOS) indicated that most included studies were of moderate to high methodological quality. Of the 19 cross-sectional studies, 10 were rated as high quality and 9 as fair quality. All case-control studies and the single cohort study were rated as high quality. Detailed quality scores for each study are presented in Table 1.

**Table 1:** Quality appraisal following the Newcastle-Ottawa scale (NOS)

Study (Author, Year)	Study Design	Selection (max 4)	Comparability (max 2)	Outcome/Exposure (max 3)	Total Score (9)	Quality
VanWormer et al. 2025 [26]	Case-control	★★★★	★★	★★	8	High Quality
Lim et al. 2025 [30]	Cohort	★★★	★★	★★	7	High Quality
Radujković et al. 2024 [31]	Case-control	★★★★	★★	★★★	9	High Quality
Schimmel et al. 2011 [48]	Case-control	★★★	★	★★★	7	High Quality

Study (Author, Year)	Study Design	Selection (max 5)	Comparability (max 1)	Outcome/Exposure (max 3)	Total Score (9)	Quality
Schulze et al. 2024 [32]	Cross-sectional	★★★	★	★★	6	Fair quality
Tian et al. 2023 [25]	Cross-sectional	★★★	★	★★★	7	High Quality
Taques et al. 2023 [33]	Cross-sectional	★★★★★	★	★★	8	High Quality
Pien et al. 2023 [24]	Cross-sectional	★★★	★	★★	6	Fair quality
Ying Li et al. 2023 [34]	Cross-sectional	★★★★	★	★★	7	High Quality
Lazureanu et al. 2022 [35]	Cross-sectional	★★★★	★	★★★	8	High Quality
Huang et al. 2022 [36]	Cross-sectional	★★★	★	★★	4	Fair quality
Schmalz et al. 2021 [37]	Cross-sectional	★★★	★	★★	6	Fair quality
Lawal et al. 2021 [38]	Cross-sectional	★★★	-	★★	5	Fair quality
Molania et al. 2021 [23]	Cross-sectional	★★★★	★	★★★	8	High Quality
Schmalz et al. 2020 [39]	Cross-sectional	★★	★	★★	6	Fair quality
Tew et al. 2020 [40]	Cross-sectional	★★★	-	★★	5	Fair quality
De Angelis et al. 2018 [41]	Cross-sectional	★★★★	★	★★	7	High Quality
da Silva et al. 2015 [42]	Cross-sectional	★★	-	★★	4	Fair quality
Jang et al. 2015 [43]	Cross-sectional	★★★★	★	★★	7	High Quality
Segura-Saint-Gerons 2012 [44]	Cross-sectional	★★★	★	★★	6	Fair quality
McGrath et al. 2009 [45]	Cross-sectional	★★★	-	★★	5	Fair quality
Hunter et al. 2006 [46]	Cross-sectional	★★★	★	★★★	7	High Quality
McMillan et al. 2005 [47]	Cross-sectional	★★★	★	★★★	7	High Quality

### Characteristics of included studies

The 23 included studies comprised 19 cross-sectional studies [23–25, 32–44, 46, 47], three case-control studies [26, 31, 48], and one cohort study [30]. Studies were conducted across a wide range of geographical regions, including North America, Europe, Asia, Africa, and South America, reflecting diverse healthcare settings and populations. The study populations included patients with stroke, heart failure, heart transplantation, left ventricular assist devices, and general cardiovascular disease. Sample sizes varied substantially, ranging from small clinical samples to large population-based cohorts. A summary of study characteristics is provided in Table 2.

### Overall impact of cardiovascular conditions on OHRQoL

Across the included studies, individuals with cardiovascular disease or a history of stroke generally reported impaired OHRQoL (e.g., Taques et al. (2023); Lazureanu et al. (2022); Jang et al. (2015); Schimmel et al. (2011) [33, 35, 43, 48]. Although not all studies demonstrated statistically significant differences between patient groups and controls, most reported higher OHRQoL impact scores compared with reference populations or normative values. Poorer OHRQoL was particularly evident among patients with greater disease severity, functional limitations, or concurrent oral conditions. Limited longitudinal evidence further suggested that poor OHRQoL was associated with the presence of cardiovascular disease over time, supporting a meaningful relationship between cardiovascular health and perceived oral health status.

### OHRQoL instruments and measurement properties

A variety of validated instruments were used to assess OHRQoL [23, 25, 34, 35]. The most frequently applied tool was the OHIP-14, followed by the GOHAI, with fewer studies using shortened or extended versions of the OHIP (e.g., OHIP-7, OHIP-49). Reported mean OHRQoL scores varied widely across study populations, reflecting differences in disease type, severity, and oral health status. Only a subset of studies reported internal consistency reliability; where reported, Cronbach’s alpha values generally indicated good to excellent reliability, supporting the validity of the applied instruments.

### Factors associated with poorer OHRQoL

#### Behavioral and oral hygiene–related factors

Behavioral factors demonstrated a consistent association with OHRQoL (e.g., findings reported by Jang et al.; Taques et al.; Lazureanu et al.) [33, 35, 43]. Poor oral hygiene practices, including infrequent tooth brushing, lack of interdental cleaning, and irregular dental visits, were repeatedly associated with worse OHRQoL. Conversely, better self-care behaviors were linked to lower OHRQoL impact, suggesting a protective role of oral hygiene practices in cardiovascular and stroke populations.

#### Oral and periodontal clinical indicators

Clinical oral health indicators showed strong and consistent associations with OHRQoL (e.g., Hunter et al.; Schimmel et al.; Taques et al.) [33, 46, 48]. Higher DMFT scores, greater tooth loss, presence and severity of periodontal disease, gingival inflammation, and xerostomia were uniformly linked to poorer OHRQoL. Studies that assessed periodontal status in detail reported particularly large effects, with advanced periodontal disease and the coexistence of cardiovascular and periodontal conditions associated with the greatest impairment, especially in physical pain and functional domains.

#### Cardiovascular-related and functional factors

Disease-related and functional parameters were important determinants of OHRQoL (e.g., Lazureanu et al.; Schimmel et al.; Jang et al.) [35, 43, 48]. Greater cardiovascular disease severity, stroke-related disability, reduced activities of daily living, swallowing dysfunction, and impaired motor or masticatory function were consistently associated with worse OHRQoL. Biomarkers of cardiac dysfunction and measures of neurological impairment further supported the link between systemic disease burden and reduced oral health–related quality of life.

#### Sociodemographic and psychosocial factors

Sociodemographic and psychosocial characteristics were also associated with OHRQoL, although findings were more heterogeneous (e.g., Taques et al. (2023); Jang et al. (2015); Hunter et al. (2006)) [33, 43, 46]. Older age, lower educational level, reduced health literacy, depression, and poorer self-rated general health were frequently linked to worse OHRQoL. Gender differences were inconsistently reported, but several studies indicated poorer perceived OHRQoL among female participants.

**Table 2:** General information on the included studies

Author (Year)	Country	Study Design	Study Population and Characteristics	Measurements		Main Findings	Methodological Details and Instrument Validity/Reliability
				Subjective Assessment	Objective Assessment		
VanWormer et al. 2025 [26]	United States	Case-control	410 adults (135 HF cases, 275 HF-free controls) aged 35–84 years Mean age: 69 years; 60% male; 97% White non-Hispanic	OHIP-5 Oral hygiene behaviors Last dental visit Smoking status, education level	Confirmed HF diagnosis using validated algorithm and Framingham criteria BMI, Medicaid status, ambulatory care visits (past 3 years) Type 2 diabetes status	Excellent oral hygiene was associated with significantly lower odds of heart failure compared to fair/poor hygiene [aOR = 0.45 (95% CI: 0.21–0.95), *p* = 0.037] Recent dental visit (< 2 years) also linked to lower HF odds compared to visits ≥ 2 years ago [aOR = 0.35 (95% CI: 0.19–0.63), *p* = 0.001] No significant association found between OHRQoL (OHIP-5) score and heart failure status (*p* = 0.988)	Matched case-control (age, sex); response rates: 67% in cases and 74% in controls; OHIP-5 used; validation [49], reference not explicitly reported by the authors
Lim et al. 2025 [30]	Korea	Observational Longitudinal Cohort Study	5,413 older adults (aged ≥ 55 years) from KLoSA, surveyed in 2018 and 2020	GOHAI Self-reported diagnosis of cardiovascular disease (CVD), including heart disease and suspected stroke or TIA	Indirect clinical indicators included as control variables: age, hypertension, diabetes, obesity, smoking, alcohol consumption, and household status	Poor OHRQoL (GOHAI < 40) was significantly associated with increased odds of having CVD (OR = 1.13, 95% CI: 1.07–1.19, *p* < 0.001). The association was stronger in females (OR = 1.36) compared to males (OR = 1.12), suggesting sex-specific differences in the relationship between oral health and cardiovascular conditions.	longitudinal panel study; follow-up: 24 months; GOHAI used (Korean validated version) [50]; internal consistency not reported by the authors; loss to follow-up: not mentioned
Radujković et al. 2024 [31]	Croatia	Comparative observational study (case-control)	42 participants (21 stroke patients in hospital group, 21 controls) Mean age: (Control group = 65.76 ± 13.30; Hospital group = 65.43 ± 13.04)	OHQL questionnaire	PPD, CAL, FMBS, FMPS, BOP, PI, PISA, PESA cIMT CRP HDL, LDL, Cholesterol, Triglycerides	Hospital group had significantly worse OHQL (*p* = 0.003) and FMPS (*p* = 0.005); Control group had higher FMBS (*p* = 0.045); PISA was negatively correlated with smoking, LDL, and cholesterol; positively correlated with FMBS, CAL, and PPD. PESA was positively correlated with the number of teeth and negatively with age, OHQL, and left cIMT. No significant group differences in PISA or PESA.	Matched case-control (Age, sex, number of participants); response rates: not mentioned; OHQL questionnaire used; specific instrument version, language, and validation details not reported by the authors
Schulze et al. 2024 [32]	Germany	Cross-sectional	58 CHF patients; equally divided into 2 groups: 29 with periodontitis (10 women, 19 men) and 29 without periodontitis (18 women, 11 men); aged 18–80 years; nonsmokers; >10 teeth Mean age: (PG = 70.69 ± 6.6; NPG = 64.21 ± 10.4)	OHIP-14 MEDAS PHQ	PSR, EF, systolic/diastolic function), NT-proBNP, troponin T, hsCRP, IL-6, leukocyte count, cholesterol, anthropometrics	The mean OHIP-14 score in the PG was 5.26 ± 7.16 vs. 3.52 ± 4.35 in the NPG (*p* < 0.87). Patients with periodontitis (PG) showed significantly higher cardiac biomarkers (NT-proBNP and hs-troponin T) compared to non-periodontitis patients. Although direct group comparisons of EF and E/A ratio were not reported as significant, poorer periodontal status correlated with reduced cardiac function indicators. No significant differences were observed between groups in inflammatory markers (CRP, IL-6), physical performance, or depression scores (PHQ-9, PHQ-2).	Convenience sample; OHIP-14 used (German validated version) [51]; internal consistency not reported by the authors; response rate not mentioned; no adjustment for confounders.
Tian et al. (2023) [25]	China	Cross-sectional	120 hospitalized post-stroke patients aged ≥ 60 years MMSE ≥ 24, able to read and communicate Two groups: with dysphagia (*n* = 56) and without dysphagia (*n* = 64) Mean age: (non-dysphagia = 67; Dysphagia = 64.21 ± 10.4)	GOHAI EAT-10	OHAT BI mRS NIHSS	Participants with dysphagia had significantly poorer oral health and OHRQoL. OHAT scores: Dysphagia group = 8.89 (3.07) vs. non-dysphagia = 5.28 (2.33) GOHAI scores: Dysphagia = 39.62 (7.52) vs. non-dysphagia = 43.96 (6.23) They also had less frequent tooth brushing. Logistic regression showed that NIHSS score and OHAT score were significant predictors of dysphagia.	Purposive sampling; GOHAI used (Chinese validated version [52], Cronbach’s α = 0.81); response rate not reported; adjustment for confounders not reported.
Taques et al. (2023) [33]	Brazil	Cross-sectional	125 patients with circulatory system diseases under outpatient care; aged ≥ 18; minimum 6 teeth; no medication changes in last 2 months	OHIP-14 SF-36	Clinical periodontal exam (biofilm, bleeding on probing, suppuration, probing depth, gingival recession, clinical attachment level)	The mean OHIP-14 score: 7.14 ± 5.91 The older people and men had greater periodontal disease severity. The older people had lower scores in SF-36 domains “Functional Capacity” and “Physical Aspects”. The “Pain” domain (SF-36) scored lowest in those with health/gingivitis. OHIP-14 showed the highest impact in “Psychological Discomfort” but no significant differences by age/gender or disease severity. Total OHIP score: 7.14 ± 5.91 No correlation found between periodontal severity and QoL instruments.	Convenience sample; OHIP-14 used [53]; psychometric properties (Cronbach’s α) not reported in this study; response rate: Not mentioned; adjustment for confounders.
Pien et al. (2023) [24]	Taiwan	Cross-sectional	Data from the 8th wave of the Taiwan Longitudinal Study on Aging (TLSA); 7702 adults aged 50 and over; mean age: 63.3 ± 9.6 years; 52.9% female	OHIP-7T self-reported physician-diagnosed stroke Health literacy ADL SPMSQ CESD-SF	n/a	The mean OHIP-7T The score was 3.12 ± 4.53. Stroke history (OR = 2.14), low health literacy (OR = 2.50), ≥ 1 ADL disability (OR = 2.71), and depression (OR = 3.28) were significantly associated with poor OHRQoL. Cognitive impairment was not significantly associated.	Convenience sample; OHIP-7T used (Taiwan validated version [54], to assess the OHRQoL of older adults, psychometric properties (Cronbach’s α) not reported in this study; response rate 93.67%; adjustment for confounders.
Ying Li et al. (2023) [34]	China	Cross-sectional	308 ischemic stroke patients, aged 41–91, ≥ 4 natural teeth, no severe comorbidities or communication problems	OHIP-14 TPB Questionnaire	MoCA mRS	The mean OHIP-14 score was 13.3 ± 7.6. Cognitive function and age had direct effects on OHRQoL. Final SEM fit was acceptable; 32% of intention variance and 26% of OHRQoL variance explained.	Convenience sample; OHIP-14 used (Chinese validated version [55], Cronbach’s α = 0.89); response rate 96.2%; adjustment for confounders.
Lazureanu et al. (2022) [35]	Romania	Cross-sectional	221 adults, CVD = 147, Age = 61.9 ± 15, M = 108, F = 113	OHIP-14 Secondary questionnaire (demographic data, oral hygiene habits, alcohol/smoking, physical activity)	Clinical oral examination (Included assessment of periodontal status using BOP, PD, CAL, and oral hygiene indices (Debris Index, Calculus Index, OHI-S) Medical record data and physical measurements	Mean OHIP-14 score: 12.5 ± 9.1. Patients with periodontal disease had significantly worse OHRQoL (*p* < 0.001, very large effect size, η² = 0.937). Patients with both cardiovascular and periodontal disease had the worst OHRQoL. Most affected domains: physical pain, physical disability, functional limitation. No significant differences in OHRQoL based on sex or urban/rural residency. Multiple regression showed periodontal disease, poor oral hygiene, and higher BMI were significant predictors of lower OHRQoL (*p* < 0.001). Psychological domains were less affected than physical ones.	Convenience sample; OHIP-14 used (Romanian validated version [56], Cronbach’s α = 0.88); response rate: Not mentioned; adjustment for confounders.
Huang et al. (2022) [36]	China	Cross-sectional	281 hospitalized stroke patients (219 males, 62 females), aged ≥ 18 years, conscious, stable, and able to communicate. Mean age: 57.94 ± 10.94	OHIP-14 Oral health knowledge, attitude, and practice (KAP) questionnaire Self-rated oral and general health	BI NRS 2002 WST Clinical confirmation of stroke diagnosis (CT/MRI) Demographic/clinical data (e.g., number of teeth, chronic diseases)	Mean OHIP-14 score = 8.37 ± 6.67 (highest for painful discomfort). Poor oral health knowledge (47.6% correct), low preventive practices (e.g., 9.3% floss use). OHRQoL is significantly associated with age, toothache, self-rated oral health, and dental visit history.	Convenience sample; OHIP-14 used (Chinese validated version [55], Cronbach’s α = 0.93); response rate 93.67%; adjustment for confounders.
Schmalz et al. 2021 [37]	Germany	Cross-sectional	186 cardiac pts (HTx = 104, HF = 82), Age ≈ 55 Mean age: (LVAD = 58.20 ± 9.37; HF = 58.20 ± 8.94)	OHIP-G14 SF-36	Demographics, smoking status, BMI, ejection fraction, comorbidities, oral exam (DMFT, PPD, CAL)	The OHIP G14 sum score was comparable between both groups, showing mean values of 3.5 ± 6.8 (LVAD) and 2.9 ± 5.4 (HF; *p* = 0.70). No significant difference in OHIP G14 scores between groups. LVAD patients had worse physical functioning and social functioning (SF-36). Oral health parameters were similar between groups. DMFT and the number of teeth correlated with OHRQoL and HRQoL in both groups.	Convenience sample; OHIP G14 used (German validated version [51], psychometric properties (Cronbach’s α) not reported in this study; response rate: Not mentioned; adjustment for confounders.
Lawal et al. 2021 [38]	Nigeria	Cross-sectional	60 stroke survivors, aged ≥ 18, mean age 55.7 ± 12.9	OHIP-14	OHI-S (Simplified Oral Hygiene Index), MAS (Modified Ashworth Scale for spasticity), ARAT (Action Research Arm Test for motor function)	Mean OHIP-14 score = 2.87 ± 0.88. Poor oral hygiene was significantly associated with dominant arm involvement (*p* = 0.013) and greater muscle spasticity. Better upper extremity mobility correlated with improved oral hygiene (*r* = −0.423, *p* < 0.001). Oral health had a weak impact on QoL in 86.7% of participants, with physical pain being the most reported oral problem.	Purposive sample; OHIP-14 used [53], psychometric properties (Cronbach’s α) not reported in this study; response rate: Not mentioned; no adjustment for confounders.
Molania et al. 2021 [23]	Iran	Cross-sectional	240 CVD patients (148 males, 92 females), aged 29–88 years (mean: 59.34 ± 18)	OHIP-14	DMFT, Plaque Index (PI), Gingival Index (GI), Sulcus Bleeding Index (SBI), xerostomia assessment, denture use	42.5% had xerostomia, significantly associated with age (*P* = 0.002), DMFT (*P* < 0.001), and “other systemic diseases” (*P* = 0.026). Mean OHIP-14 score: 21.34 ± 17.4 (ADD), 6.11 ± 5.07 (SC). Poorer OHRQoL linked to xerostomia (*P* < 0.001), higher DMFT (*P* < 0.001), and removable denture use (*P* = 0.009). Regression showed main determinants of poor OHRQoL: xerostomia, DMFT, SBI, denture use, and gender (females worse).	Random sample; OHIP-14 used (Farsi validated version [57], Cronbach’s α = 0.953; response rate: Not mentioned; adjustment for confounders.
Schmalz et al. 2020 [39]	Germany	Cross-sectional	186 participants: HTx: 104 Age: 55.26 ± 12.16 HF: 82 Age: 54.90 ± 11.14	OHIP-G14 SF-36	Clinical oral exam (DMF-T, PPD, CAL), treatment need assessment	OHIP- G14 score: 6.58 ± 6.40 in the HTx group and 5.54 ± 5.47 in the HF group. No clinically significant difference in overall OHRQoL was found between the HTx and HF groups. The oral function subdomain score was slightly better in the HF group (*p* = 0.04), but this difference was not clinically relevant. In the HF group, higher periodontal treatment need and poorer physical and mental HRQoL (SF-36 PCS and MCS scores) were significant predictors of worse OHRQoL. Smoking also emerged as a negative predictor of OHRQoL in the HF group.	Convenience sample; OHIP-G14 used (German validated version [58], psychometric properties (Cronbach’s α) not reported in this study; response rate: Not mentioned; adjustment for confounders.
Tew et al. 2020 [40]	Malaysia	Cross-sectional	31 stroke survivors (22 males, 9 females), mean age: 62 ± 10.01 years	OHIP-14 EQ-5D-5 L (General Health Status)	Sociodemographic data, comorbidities, and oral hygiene practices	Higher education level was significantly associated with poorer OHRQoL (*p* = 0.028). No significant effect of gender, age, comorbidities, or oral hygiene practices on OHRQoL. Most patients reported minimal problems, except food impaction (61.3%) affected OHRQoL.	Convenience sample; OHIP-14 used (Malaysian validated version [59]), psychometric properties (Cronbach’s α) not reported in this study; response rate: Not mentioned; no adjustment for confounders.
De Angelis et al. 2018 [41]	Italy	Cross-sectional	533 older adults (308 females, 225 males), aged 65–98 years mean age: 73.93 ± 7.8	GOHAI	DMFT index (CPI), presence of fixed/removable dentures; sociodemographic and medical history data	GOHAI score: 15.24 ± 8.88. 69.8% had cardiovascular disease (CVD). Patients with CVD had significantly higher CPI, more missing teeth, and fewer filled teeth (*P* < 0.05). A higher number of missing teeth (≥ 18) was associated with a 2.5 times greater risk of CVD (*P* < 0.05). GOHAI scores decreased with age; women had lower scores (worse perception), though not statistically significant. Logistic regression showed CPI, number of missing and filled teeth were independently associated with CVD.	Convenience sample; GOHAI used [60], psychometric properties (Cronbach’s α) not reported in this study; response rate: Not mentioned; adjustment for confounders.
da Silva et al. 2015 [42]	Brazil	Cross-sectional	27 stroke survivors with complete or partial hemiparesis (brachial or crural predominance); mean age: 60.5 ± 12.7 years; both genders	OHIP-14 SSQOL-Brazil	n/a	OHIP-14 score: 4.63 ± 6.60. Reduced upper limb function was significantly associated with worse OHRQoL. Strongest correlation observed with physical pain (*r* = − 0.707); overall, oral health had a weak impact on QoL in most participants.	Convenience sample; OHIP-14 used [61], psychometric properties (Cronbach’s α) not reported in this study; response rate: Not mentioned; no adjustment for confounders.
Jang et al. 2015 [43]	Korea	Cross-sectional	549 stroke survivors, Age = ≥ 60: 473, M = 329, F = 220 Mean age:	OHIP-14 demographics; stroke/ADL measures; oral health behaviors; subjective oral and general health	Demographics, stroke duration, disability (modified Rankin), ADL (Barthel Index), BMI, oral health behaviors (tooth brushing frequency, floss use, denture use), missing teeth	OHIP-14 score: 33.0 ± 9.0; worse OHRQoL with older age, poor subjective health, more severe disability, lower ADL, unhealthy oral status, no floss, more missing teeth, no denture; key predictors: ADL, brushing frequency, subjective health, oral health status	Convenience sample; OHIP-14 used (Korean validated version [62], psychometric properties (Cronbach’s α) not reported in this study; response rate: Not mentioned; adjustment for confounders.
Segura-Saint-Gerons 2012 [44]	Spain	Cross-sectional	150 patients Heart transplant patients (≥ 18 years), visiting the hospital for clinical follow-up 54.94 years (SD 14.56; range 19–79) 118 men (78.7%), 32 women (21.3%)	OHIP-49	Collected demographic data, habits (oral hygiene frequency, dental visits), type of transplant, years since transplantation	OHIP-49 score: 24.43; highest domains: physical pain, functional limitation; women had significantly worse OHRQoL (*p* = 0.008); predictors: gender, brushing frequency, dental visits.	Convenience sample; OHIP-49 (Spanish validated version [63]), psychometric properties (Cronbach’s α) not reported in this study; response rate: Not mentioned; adjustment for confounders.
Schimmel et al. 2011 [48]	Switzerland	Case-control	PG: 31 stroke patients (mean age: 69.0 ± 12.7), CG: 24 non-stroke subjects (mean age: 68.8 ± 10.8), matched for age, gender, dental state	OHIP-49	NIHSS (stroke severity), Masticatory efficiency (UF), Maximum Lip Force (MLF), Eichner classification, Occlusal units, Prosthodontic rehabilitation type	PG had significantly lower OHIP-49 score: PG = 18.8 ± 15.5 vs. CG = 12.3 ± 17.7, *p* = 0.0131) Chewing efficiency (UF) and MLF are significantly lower in PG UF was the main predictor of lower OHRQoL in PG In CG, age and the Eichner group influenced the OHIP score	Matched case-control (age, sex); response rates: not mentioned; OHIP-49 used [61] (psychometric properties (Cronbach’s α) not reported in this study
McGrath et al. 2009 [45]	Hong Kong	Longitudinal observational study	161 stroke patients at baseline, 65 patient–proxy pairs at 6-month follow-up; mean patient age: 68.3 years; predominantly male patients (72.3%) and female proxies (72.3%)	GOHAI SF-12	GOHAI (proxy) SF-12 (proxy)	Significant improvements in OHRQoL (GOHAI, SF-12) over time from patient self-reports; moderate to excellent agreement at baseline (ICC = 0.69–0.86), weaker at 6 months (ICC = 0.28–0.60); largest discrepancies in mental health (MCS-12); highlights limitations of proxy assessments for subjective outcomes	Consecutive sample; GOHAI (Chinese validated version [52], psychometric properties (Cronbach’s α) not reported in this study; response rate: Not mentioned; no adjustment for confounders.
Hunter et al. 2006 [46]	Scotland	Cross-sectional	41 stroke survivors (mean age 69 ± 9.8 years), attending a 1-year post-stroke outpatient review; 21 females, 20 males; 23 dentate, 18 edentulous; various levels of post-stroke disability	OHIP-14	Clinical oral exam: number of teeth, plaque index, denture hygiene, periodontal condition.	37% reported difficulty cleaning teeth, significantly associated with disability (*p* = 0.015). Functional limitation was the most frequent OHIP dimension. Edentulous subjects reported more impacts, but the difference in OHIP score was not significant. Stroke-related disability led to reduced dental visits post-stroke.	Convenience sample; OHIP-14 used, psychometric properties (Cronbach’s α) not reported in this study; response rate: Not mentioned; no adjustment for confounders.
McMillan et al. 2005 [47]	China	Cross-sectional	43 stroke patients, Age = 73.9 ± 6.1, M = 26, F = 17	GOHAI SF-36	DMFT index	The overall average score of GOHAI was 51.09 ± 6.20. The mean GOHAI score for the stroke group was 52 and 54 for the comparison group, with no significant difference, although more stroke survivors had difficulty speaking compared to the comparison group. There were some OHR-QoL disorders.	Consecutive sample; GOHAI (Chinese validated version [52]), psychometric properties (Cronbach’s α) not reported in this study; response rate: Not mentioned; adjustment for confounders.

#### Objective oral health measures

Objective oral health assessments supported the subjective OHRQoL findings (e.g., Hunter et al.; Schimmel et al.; Taques et al.) [33, 46, 48]. Higher DMFT scores, increased periodontal treatment needs, poorer plaque and bleeding indices, and reduced chewing efficiency were generally associated with worse OHRQoL. Although the magnitude of associations varied across studies, the direction of effects was consistent, reinforcing the clinical relevance of objective oral health status in shaping patients perceived quality of life. Detailed oral health indicators are summarized in Table 3.

**Table 3:** Primary outcomes related to oral condition and oral health parameters across the included studies

Author, year	Tooth loss, remaining teeth, dentures	Dental diseases, caries, and dental treatment need	Oral hygiene indices
VanWormer et al. 2025 [26]	Not reported	Not reported	Not reported
Lim et al. 2025 [30]	Not reported	Not reported	Not reported
Schulze et al. 2024 [32]	Number of teeth: 23.7 (PG) vs. 25.1 (NPG), n. s	Not reported	Not reported
Radujković et al. 2024 [31]	Mean number of teeth (Control: 22.05, Hospital: 20.10, *p* = 0.136).	Not reported	FMPS (Full-Mouth Plaque Score) Control: 16.83 ± 7.17, Hospital: 27.57 ± 15.13 FMBS (Full-Mouth Bleeding Score) Control: 10.17 ± 6.10, Hospital group: 6.42 ± 5.62
Tian et al. 2023 [25]	Not reported	Not reported	The non-dysphagia group brushed their teeth more frequently than the dysphagia group (*p* < 0.05). OHAT score: Non-dysphagia group: 5.28 ± 2.33 Dysphagia group: 8.89 ± 3.06
Taques et al. 2023 [33]	Number of missing teeth > 9: 33% (Mean 9.2 ± 7.5) Number of missing teeth in older people (≥ 60 years): 45% (12.6 ± 6.7) Number of missing teeth in < 60 years: 28% (7.8 ± 7.4)	Gingivitis/Healthy (Score 1): 9% Periodontitis Stage 1–2 (Score 2): 50% Periodontitis Stage 3–4 (Score 3): 41% Probing depth ≥4 mm: 14% of sites Clinical attachment level ≥3 mm: 19% of sites	Biofilm: 68% Bleeding on probing: 48% Suppuration: 5%
Pien et al. 2023 [24]	Not reported	Not reported	Not reported
Ying Li et al. 2023 [34]	Not reported	Not reported	Not reported
Lazureanu et al. 2022 [35]	Missing teeth 9.2 ± 1.32	Periodontal disease Stage I–IV: 59.3% (Stage III–IV: 10.4%) Strong impact of periodontal disease on OHRQoL (η²*p* = 0.937)	Dental plaque 51.6% Brushing ≥ 2/day: 70.1%; once/day: 28.5%; rarely: 1.4% Flossing 33.0%, Mouthwash use 43.9% Dental scaling: Never 74.6%, once/year 22.2%, twice/year 3.2%
Huang et al. 2022 [36]	Denture use Partial: 29.9% All: 3.2% Number of teeth < 10: 8.9% > 20: 76.9% Number of missing teeth > 9: 11.4%	Oral odor 51.2% Dry mouth 74.0% Gingival bleeding 92.5% Tooth decay 59.8% Toothache 86.5%	Brushing ≥ 2/day: 49.1% Floss use: 9.3% Toothpick use: 34.9%; rinsing with tap water: 92.5%
Schmalz et al. 2021 [37]	Denture Use Partial Denture: 29.9% Full Denture: 3.2% Number of Teeth < 10 Teeth: 8.9% > 20 Teeth: 76.9% Remaining teeth LVAD: 18.82 ± 9.67 HF: 16.53 ± 8.75 Remaining Molar/Premolar Teeth LVAD: 9.72 ± 5.56 HF: 8.00 ± 5.41	DMF-T LVAD: 17.45 ± 7.66 HF: 18.21 ± 7.11 D-T LVAD: 0.36 ± 1.33 HF: 0.50 ± 1.56	Not reported
Lawal et al. 2021 [38]	Not reported	Not reported	OHI-S: 2.42 ± 1.34 (Male), 2.22 ± 0.91 (Female)
Molania et al. 2021 [23]	Removable partial denture: 16.76 ± 16.57	Xerostomia: 3.37 ± 3.15 DMFT: 20.96 ± 8.26	PI: 1.98 ± 0.62 GI: 1.68 ± 0.75 SBI: 0.78 ± 0.39
Schmalz et al. 2020 [39]	M-T HTx:6.90 ± 7.27 HF:7.32 ± 7.64	DMF-T HTx: 16.08 ± 7.11 HF=:16.90 ± 6.66 Dental treatment needs HTx: 16.3% HF: 17.1%	Not reported
Tew et al. 2020 [40]	Not reported	Not reported	Not reported
De Angelis et al. 2018 [41]	Dentures: 20.9% Tooth Loss Missing < 5 teeth: 27.8% Missing > 18 teeth: 22.9% Remaining Teeth > 18 teeth remaining: 58.1%	Dental Treatment Need Need for more fillings: 33.2% Need for full dentures: 16.5%	CPI Score: 18.3 ± 7.5
da Silva et al. 2015 [42]	Not reported	Not reported	Not reported
Jang et al. 2015 [43]	Dentures: 46.9% missing tooth 1–8: 24.5%	Not reported	Frequency of tooth brushing per day 1 time per day: 31.7% 2 or more times per day: 54.6%
Segura-Saint-Gerons 2012 [44]	Fixed dentures: 13 patients (59.3%) Removable resin dentures: 32 patients (21.3%) Removable skeletal dentures: 16 patients (10.7%)	Not reported	Frequency of tooth brushing per day 1 time per day: 28.7% 2 times per day: 30.7%
Schimmel et al. 2011 [48]	Mean number of remaining teeth Patient Group (PG): 18.8 ± 8.9 Control Group (CG): 18.2 ± 9.3 Number of occlusal units (OU) Patient Group (PG): 4.3 ± 4.0 Control Group (CG): 5.5 ± 5.1 Removable partial denture use Patient Group (PG): 19.4% Control Group (CG): 12.5%	Not reported	Chewing efficiency (UF score) Patient Group (PG): 0.0901 ± 0.0488 (↓ significantly) Control Group (CG): 0.0442 ± 0.0304 (↑ better) Lip force (MLF –small/medium/large) Patient Group (PG): Lower in PG (e.g., 5.29 N, 6.70 N, 8.68 N) Control Group (CG): Higher in CG (e.g., 6.90 N, 8.47 N, 10.17 N)
McGrath et al. 2009 [45]	Not reported	Not reported	Not reported
Hunter et al. 2006 [46]	Dentate: 23 (56%), of which 70% male Mean no. of teeth in dentate: 17 Partial denture: 12/23 dentate Edentulous: 18 (mostly female) Full upper & lower dentures in all edentulous areas	80% of dentate had a CPI score of 1 or 2 in ≥ 1 sextant → indicated treatment need	Brushing once/day: 51% Twice/day: 32% Difficulty cleaning teeth: 37% overall
McMillan et al. 2005 [47]	Missing: 18 ± 1.07 Removable denture: Stroke: 40.5%; Comparison: 51.2% Complete denture: Stroke: 20.9%; Comparison: 16.3%	No. of teeth: Stroke: 14.6 ± 10.8; Comparison: 17.2 ± 9.9 Decayed: Stroke: 2.7 ± 3.7; Comparison: 1.1 ± 1.6 Missing: Stroke: 18.0 ± 10.7; Comparison: 15.3 ± 9.6 Filled: Stroke: 0.9 ± 2.1; Comparison: 1.9 ± 2.5 DMF-T: Stroke: 21.6 ± 9.7; Comparison: 18.3 ± 9.6	Brushing once/day: 51% Twice/day: 32% Difficulty cleaning teeth: 37% overall

*DMF-T* decayed, missing, and filled-tooth index, *M-T* missing teeth, *D-T* decaying teeth, *F-T* filled teeth, *GI* gingival index, *SBI* Sulcus bleeding index, *PI* plaque index, *FMPS* Full-Mouth Plaque Score, *FMBS* Full-Mouth Bleeding Score, *OHI-S* Simplified Oral Hygiene Index, *CPI* Community Periodontal Index, *OHAT* Oral Health Assessment Tool, *η²p* Partial Eta Squared, *Edentulous* having no natural teeth, *Dentate* having one or more natural teeth.

### Most consistently affected OHRQoL domains

Detailed domain- and item-level OHRQoL findings across studies are summarized in Table 4.

Synthesis of domain-level findings revealed a consistent pattern in the OHRQoL dimensions most affected among patients with cardiovascular disease and stroke (as reported by Taques et al. (2023); Lazureanu et al. (2022); Hunter et al. (2006); Schimmel et al. (2011)) [33, 35, 46, 48]. Physical pain and functional limitation were the most frequently and severely impacted domains across studies, regardless of the specific OHRQoL instrument used. Psychosocial discomfort, including psychological distress and self-consciousness related to oral conditions, was also commonly reported, particularly among stroke survivors and patients with coexisting cardiovascular and periodontal disease. In contrast, social disability and handicap domains were less consistently affected and generally showed lower impact scores.

Overall, these findings indicate that the physical and functional dimensions of OHRQoL are disproportionately affected in cardiovascular and stroke populations.

**Table 4:** OHRQoL subscales in the OHIP questionnaire

Author, year	Functional Limitation	Physical Pain	Psychosocial Discomfort	Physical Disability	Psychological Disability	Social Disability	Handicap
Taques et al. 2023 [33]	0.64 ± 0.90	1.30 ± 1.03	1.66 ± 1.33	0.96 ± 1.13	1.19 ± 1.13	0.71 ± 1.05	0.68 ± 1.07
Lazureanu et al. 2022 [35]	2.15 ± 1.55	3.09 ± 1.81	1.69 ± 1.56	2.09 ± 1.98	0.82 ± 1.3	1.13 ± 1.28	1.45 ± 1.23
Lawal et al. 2021 [38]	0.65 ± 0.18	1.12 ± 0.27	0.63 ± 0.18	0.70 ± 0.19	0.27 ± 0.09	0.03 ± 0.02	0.00 ± 0.00
Jang et al., 2015 (male/female) [43]	5.7 ± 2.0/ 5.9 ± 2.1	5.2 ± 1.8/ 5.4 ± 1.9	4.7 ± 1.5/ 4.6 ± 1.5	5.2 ± 1.8/ 5.1 ± 1.9	3.8 ± 1.4/ 3.7 ± 1.4	3.8 ± 0.4/ 3.6 ± 1.4	4.7 ± 1.8/ 4.7 ± 1.9
da Silva et al. 2015 [42]	0.74 ± 1.2	0.66 ± 1.1	1.06 ± 1.3	0.57 ± 0.9	0.67 ± 1.0	0.47 ± 1.1	0.45 ± 1.0
Schimmel et al. 2011 (PG/CG) [48]	4.2 ± 3.7 2.7 ± 2.9	4.2 ± 3.6 2.8 ± 4.2	3.0 ± 2.8 1.7 ± 2.5	3.1 ± 3.8 1.9 ± 3.6	1.9 ± 2.1 1.5 ± 2.7	0.8 ± 1.6 1.0 ± 2.7	1.6 ± 2.1 0.8 ± 1.7
Tew et al. 2020 [40]	OHIP Q1: 5(16.1%) OHIP Q2: 4(12.9%)	OHIP Q3: 4(12.9%) OHIP Q4: 2(6.5%)	OHIP Q5: 10(32.3%) OHIP Q6: 1(3.2%)	OHIP Q7: 6(19.4%) OHIP Q8: 0(0.0%)	OHIP Q9: 3(9.7%) OHIP Q10: 1(3.2%)	OHIP Q11: 2(6.5%) OHIP Q12: 1(3.2%)	OHIP Q13: 5(16.1%) OHIP Q14: 2(6.5%)
Huang et al. 2022 (Items) [36]	OHIP Q1: 5(1.8%) OHIP Q2: 4(1.4%)	OHIP Q3: 38(13.5%) OHIP Q4: 28(10%)	OHIP Q5: 5(1.8%) OHIP Q6: 1(0.4%)	OHIP Q7: 7(2.5%) OHIP Q8: 3(1.1%)	OHIP Q9: 3(1.1%) OHIP Q10: 15%	OHIP Q11: 0 OHIP Q12: 1(0.4%)	OHIP Q13: 1(0.4%) OHIP Q14: 1(0.4%)
Hunter et al. 2006 (Items) [46]	OHIP Q1: 32% OHIP Q2: 24%	OHIP Q3: 10% OHIP Q4: 27%	OHIP Q5: 29% OHIP Q6: 5%	OHIP Q7: 7% OHIP Q8: 24%	OHIP Q9: 7% OHIP Q10: 15%	OHIP Q11: 5% OHIP Q12: 0%	OHIP Q13: 7% OHIP Q14: 0%

## Discussion

This systematic review provides a comprehensive synthesis of current evidence on oral health-related quality of life (OHRQoL) among individuals with cardiovascular diseases (CVD) and post-stroke conditions. The findings reveal considerable heterogeneity in OHRQoL outcomes, influenced by the type of underlying cardiovascular condition, demographic and clinical characteristics, oral health status, and psychosocial factors.

While most studies included in this review indicate that systemic cardiovascular conditions such as heart failure, left ventricular assist device implantation [37], and heart transplantation [39] are associated with impaired OHRQoL, particularly in physical pain and psychosocial discomfort domains, evidence regarding heart failure remains inconsistent. Some studies reported significant OHRQoL impairments among patients with advanced or chronic heart failure, whereas others found no clear association [26], potentially due to differences in disease severity, study design, and duration of follow-up.

The OHIP-14 [23, 32–36, 38, 40, 42, 43, 46], OHIP-G14 [37, 39], and GOHAI [25, 30, 41, 45, 47] were the primary tools used to assess OHRQoL. In contrast, the GOHAI focuses primarily on functional and pain-related aspects and may be less sensitive to psychosocial dimensions, and variability in instrument selection limits comparability across studies, underscoring the need for greater standardization in OHRQoL assessment within cardiovascular populations.

More recent investigations have reported largely similar patterns of OHRQoL impairment in cardiovascular and post-stroke populations, while benefiting from improved methodological rigor and more detailed clinical characterization. Together, these findings strengthen the overall consistency of the evidence base despite existing methodological heterogeneity.

Multiple determinants of OHRQoL were identified across studies and can be broadly categorized into clinical, behavioral, and sociodemographic factors. Clinical factors included periodontal disease severity, tooth loss, xerostomia, and comorbid conditions such as diabetes [32]. Behavioral and sociodemographic determinants included poor oral hygiene practices, smoking, older age, lower educational attainment, and limited health literacy. Together, these factors highlight the multifactorial nature of oral health challenges in cardiovascular populations [35, 64, 65].

The association between periodontal disease and systemic health conditions appears to be more pronounced in older adults, where the severity of periodontitis and biomarkers associated with chronic heart failure (CHF), such as NT-proBNP, increase with age. Age may therefore act as a significant confounder when examining the links between oral health and CHF [32, 66].

Moreover, stroke-specific findings, particularly those related to swallowing dysfunction and dysphagia, warrant more detailed consideration due to their distinct clinical and functional implications. Dysphagia has been associated not only with impaired swallowing but also with worse oral health status in post-stroke populations when compared to those without dysphagia, highlighting that oral health and self-care behaviors are uniquely affected in this subgroup [25]. Additionally, among stroke inpatients, deficits in self-care ability, including oral hygiene and nutritional intake, have been shown to co-occur with compromised oral health-related quality of life, indicating that swallowing dysfunction may exacerbate declines in both functional and psychosocial oral health outcomes [36]. From a research perspective, future studies should stratify stroke patients based on dysphagia status and explicitly integrate swallowing-related measures into analytical models to better elucidate their contribution to OHRQoL and to identify targets for tailored intervention.

Some studies reported a negative correlation between tooth loss and OHRQoL in patients with LVAD; a higher number of remaining teeth was associated with lower OHIP-G14 scores, indicating better oral health quality of life [37]. This association is consistent with broader evidence demonstrating that a greater number of remaining and functional teeth particularly occluding pairs is linked to improved OHRQoL across both physical and psychosocial domains, underscoring the clinical importance of tooth retention beyond simple tooth count measures [67, 68].

These findings are consistent with evidence reported in a recent comprehensive review that examined the links between oral health metrics and cardiovascular outcomes, particularly in the context of aging. That study highlighted associations between indicators such as periodontal disease, tooth loss, and chronic oral inflammation with cardiovascular morbidity, proposing systemic inflammation, endothelial dysfunction, and shared behavioral risk factors as plausible underlying mechanisms [69]. Situating the present results within this framework supports the relevance of oral health–related quality of life as an important dimension of cardiovascular health assessment, especially among older adults and individuals with complex medical conditions.

There is growing evidence supporting an association between oral health, particularly periodontal disease, and cardiovascular conditions. Recent high-quality reviews and critical appraisals have consistently demonstrated that periodontitis is associated with an increased risk of atherosclerotic cardiovascular disease, including coronary artery disease, heart failure, and other major cardiovascular outcomes, even after adjustment for established confounding factors such as age, smoking, and socioeconomic status [70, 71]. Several biological mechanisms have been proposed to explain this association, including systemic inflammation, endothelial dysfunction, immune-mediated pathways, and the potential translocation of periodontal pathogens contributing to vascular inflammation and atherosclerotic plaque development [71].

Nevertheless, it should be acknowledged that the majority of available evidence is derived from observational studies, which limits causal inference. While the consistency of findings across diverse populations strengthens the association, definitive conclusions regarding causality and the impact of periodontal treatment on cardiovascular outcomes remain uncertain [70, 71].

In patients with heart failure or left ventricular assist devices (LVADs), Schmalz et al. reported a direct correlation between the number of remaining teeth and improved overall health-related quality of life (OHRQoL) [37]. These findings underscore the importance of preventive dental care and early rehabilitation in the management of CVD [72, 73].

Objective measures such as the DMFT index, Plaque Index, and Gingival Index revealed suboptimal oral health across most included studies. In particular, patients with LVAD, HF, or stroke often exhibited higher plaque scores and bleeding indices. Self-reported oral hygiene behaviors, such as tooth brushing and flossing, varied widely but were generally suboptimal. Tian et al. (2023) [25] highlighted that among hospitalized post-stroke patients, those with dysphagia exhibited significantly poorer oral health behaviors, oral hygiene, and OHRQoL compared to non-dysphagia stroke patients. Swallowing function post-stroke was closely associated with oral health. These patients often experienced impaired sensory and motor functions, making self-care, including tooth brushing, more difficult [74]. Furthermore, oral care was frequently neglected in these patients, particularly when swallowing safety was compromised [75, 76].

### Strengths and limitations

A notable strength of this review is its adherence to PRISMA 2020 guidelines, ensuring transparency and methodological rigor. In addition, this review provides a focused synthesis of evidence on oral health–related quality of life (OHRQoL) among individuals with cardiovascular disease, including stroke survivors and patients undergoing cardiac or post-surgical care—an area that has received limited attention in previous reviews. The inclusion of studies from diverse countries strengthens the external validity of the findings.

Most included studies were of high or fair methodological quality, as assessed by the Newcastle-Ottawa Scale. However, several limitations must be acknowledged. First, the predominance of cross-sectional designs limits causal inference. Longitudinal studies are crucial for understanding the long-term impact of oral health on cardiovascular outcomes. Second, only a few studies reported psychometric validation of the OHRQoL instruments used. Third, there was considerable heterogeneity in outcome reporting, making quantitative synthesis unfeasible.

The findings of this review have important clinical and policy implications. Routine assessment of oral health and OHRQoL should be integrated into cardiovascular and stroke rehabilitation programs. Moreover, targeted interventions to improve oral hygiene and dental care utilization are warranted, particularly for high-risk groups such as older adults, patients with functional limitations, and those with poor health literacy. Incorporating oral health evaluations into standard cardiac care and rehabilitation protocols can significantly improve overall quality of life in these patient populations.

In addition, the exclusion of baseline data from interventional studies may have reduced the number of eligible studies. However, this decision was made to enhance methodological consistency and to avoid potential bias arising from mixed study designs. Consequently, the findings of this review should be interpreted with caution and viewed as indicative of associations rather than evidence of causality. Future research should prioritize well-designed longitudinal and interventional studies to clarify temporal relationships and underlying mechanisms linking oral health, OHRQoL, and cardiovascular outcomes.

### Future directions

Future research should prioritize longitudinal designs to examine causal pathways between cardiovascular disease progression and OHRQoL deterioration. Studies should also explore how interventions such as preventive dental care, oral hygiene education, and prosthetic use can mitigate the adverse effects of oral health on systemic conditions. Additionally, the development and validation of culturally sensitive OHRQoL instruments for use in medically compromised populations remains a research priority. Such instruments would help ensure that OHRQoL measures are meaningful and relevant across diverse populations.

## Conclusion

Oral health has a significant impact on the quality of life in patients with cardiovascular diseases. The routine integration of dental assessments, personalized interventions, and patient education into cardiac care protocols may lead to improved health outcomes. Special attention should be given to vulnerable populations, including older stroke survivors, who may experience greater oral health challenges and associated declines in well-being. These findings support the integration of oral health assessment into routine cardiovascular and stroke care, particularly for vulnerable populations such as older adults. Future longitudinal and multicenter studies are needed to better elucidate causal relationships and to assess whether oral health interventions can improve both cardiovascular outcomes and OHRQoL.

## Supplementary Information

Supplementary Material 1.

## Data Availability

The protocol for this systematic review has been registered in PROSPERO under the registration number [CRD42024609168]. The datasets used and/or analysed during the current study are available from the corresponding author upon reasonable request.

## Abbreviations

CVD: Cardiovascular Disease
QoL: Quality of Life
HRQoL: Health-Related Quality of Life
OHRQoL: Oral Health-Related Quality of Life
OHIP: Oral Health Impact Profile
GOHAI: Geriatric Oral Health Assessment Index
PRISMA: Preferred Reporting Items for Systematic Reviews and Meta-Analyses
