# Supplementary material for: Oral health-related quality of life in cardiovascular patients- a systematic review
Source: BMC Cardiovasc Disord. 2026 Feb 5;26:212. doi: 10.1186/s12872-026-05564-8 (PMC12973747; doi:10.1186/s12872-026-05564-8)
Supplement: Supplementary file 1 — Supplementary Material 1. [file 12872_2026_5564_MOESM1_ESM.docx]

Scopus:

( TITLE-ABS-KEY ( "oral health-related quality of life" ) OR TITLE-ABS-KEY ( OHRQoL ) OR TITLE-ABS-KEY ( "oral health impact profile" ) OR TITLE-ABS-KEY ( OHIP ) OR TITLE-ABS-KEY ( "General Oral Health Assessment Index" ) OR TITLE-ABS-KEY ( GOHAI ) AND TITLE-ABS-KEY ( "cardiovascular disease*" ) OR TITLE-ABS-KEY ( "heart disease*" ) OR TITLE-ABS-KEY ( "coronary artery disease" ) OR TITLE-ABS-KEY ( "myocardial infarction" ) OR TITLE-ABS-KEY ( MI ) OR TITLE-ABS-KEY ( angina* ) OR TITLE-ABS-KEY ( stroke ) OR TITLE-ABS-KEY ( CVA ) OR TITLE-ABS-KEY ( cerebrovasc* ) OR TITLE-ABS-KEY ( myocard* ) OR TITLE-ABS-KEY ( thrombo* ) OR TITLE-ABS-KEY ( "atrial fibrillat*" ) OR TITLE-ABS-KEY ( "congestive heart failure" ) OR TITLE-ABS-KEY ( CHF ) OR TITLE-ABS-KEY ( emboli* ) OR TITLE-ABS-KEY ( PAD ) OR TITLE-ABS-KEY ( PVD ) OR TITLE-ABS-KEY ( "hypertensive heart disease" ) OR TITLE-ABS-KEY ( "Coronary Heart Disease" ) OR TITLE-ABS-KEY ( "heart failure" ) OR TITLE-ABS-KEY ( "transient ischemic attack" ) OR TITLE-ABS-KEY ( "Peripheral artery disease" ) OR TITLE-ABS-KEY ( "Aortic atherosclerosis" ) ) AND ( LIMIT-TO ( LANGUAGE , "English" ) )

Pubmed

| #3 | Search: **#1 AND #2** | 101 |
| --- | --- | --- |
| #2 | Search: **(((((((((((((((((((((((((((((((((((("cardiovascular diseases"[MeSH Terms]) OR ("heart diseases"[MeSH Terms])) OR ("coronary artery disease"[MeSH Terms])) OR ("myocardial infarction"[MeSH Terms])) OR (stroke[MeSH Terms])) OR ("cerebrovascular disorders"[MeSH Terms])) OR ("heart failure"[MeSH Terms])) OR ("peripheral arterial disease"[MeSH Terms])) OR ("aortic diseases"[MeSH Terms])) OR ("atrial fibrillation"[MeSH Terms])) OR (embolism[MeSH Terms])) OR (thrombosis[MeSH Terms])) OR (hypertension[MeSH Terms])) OR ("Ischemic Attack, Transient"[MeSH Terms])) OR ("coronary heart disease"[Title/Abstract])) OR ("cardiovascular disease*"[Title/Abstract])) OR ("heart disease*"[Title/Abstract])) OR ("coronary artery disease"[Title/Abstract])) OR ("myocardial infarction"[Title/Abstract])) OR (MI[Title/Abstract])) OR (angina*[Title/Abstract])) OR (stroke[Title/Abstract])) OR (CVA[Title/Abstract])) OR (cerebrovasc*[Title/Abstract])) OR (myocard*[Title/Abstract])) OR (thrombo*[Title/Abstract])) OR ("atrial fibrillat*"[Title/Abstract])) OR ("congestive heart failure"[Title/Abstract])) OR (CHF[Title/Abstract])) OR (emboli*[Title/Abstract])) OR (PAD[Title/Abstract])) OR (PVD[Title/Abstract])) OR ("hypertensive heart disease"[Title/Abstract])) OR ("heart failure"[Title/Abstract])) OR ("transient ischemic attack"[Title/Abstract])) OR ("peripheral artery disease"[Title/Abstract])) OR ("aortic atherosclerosis"[Title/Abstract])** | 3,676,889 |
| #1 | Search: **((((("oral health-related quality of life"[Title/Abstract]) OR (OHRQoL[Title/Abstract])) OR ("oral health impact profile"[Title/Abstract])) OR (OHIP[Title/Abstract])) OR ("General Oral Health Assessment Index"[Title/Abstract])) OR (GOHAI[Title/Abstract])** | 5,858 |

Web of science:

TS=("cardiovascular disease*" OR "heart disease" OR "coronary artery disease" OR "myocardial infarction" OR "stroke" OR "cerebrovascular disorders" OR "heart failure" OR "peripheral arterial disease" OR "aortic diseases" OR hypertension OR "transient ischemic attack" OR "congestive heart failure" OR "coronary heart disease" OR "heart disease*" OR "MI" OR "angina*" OR "CVA" OR "cerebrovasc*" OR "myocard*" OR "thrombo*" OR "atrial fibrillat*" OR "CHF" OR "emboli*" OR "PAD" OR "PVD" OR "hypertensive heart disease" OR "aortic atherosclerosis") AND TS=("oral health-related quality of life" OR "OHRQoL" OR "oral health impact profile" OR "OHIP" OR "General Oral Health Assessment Index" OR "GOHAI")
